# Multi-Feature Intelligent Oral English Error Correction Based on Few-Shot Learning Technology

**DOI:** 10.1155/2022/2501693

**Published:** 2022-06-23

**Authors:** Fengxiang Zhang, Jieli Sun

**Affiliations:** ^1^College of Foreign Languages, Hebei University of Economics and Business, Shijiazhuang, Hebei 050061, China; ^2^School of Information Technology, Hebei University of Economics and Business, Shijiazhuang, Hebei 050061, China

## Abstract

The computer-aided language teaching system is maturing thanks to the advancement of few-shot learning technologies. In order to support teachers and increase students' learning efficiency, more computer-aided language teaching systems are being used in teaching and examinations. This study focuses on a multifeature fusion-based evaluation method for oral English learning, completely evaluating specific grammar, and assisting oral learners in improving their oral pronunciation skills. This study proposes an improved method based on HMM a posteriori probability scoring, in which the only standard reference model is no longer used as the basis for scoring and error determination, and instead, the average level of standard pronunciation in the entire corpus is introduced as another judgment basis, based on a preliminary study of speech recognition technology, scoring methods, and relevant theoretical knowledge of information feedback. This strategy can reduce the score limitation caused by standard pronunciation personal differences, lower the system's misjudgment rate in detecting pronunciation errors, and improve the usefulness of error correction information. An expert opinion database has been created based on the most prevalent forms of spoken pronunciation problems, which can successfully assist learners improve their spoken English level by combining the database's corrected information. Finally, this study proposes an artificial scoring system for spoken English that performs activities such as identification, scoring, error judgment, and correction opinion feedback, among others. Finally, it has been demonstrated through trials and tests that adding the average pronunciation level to the system improves the system's scoring performance and has a certain effect on increasing users' oral pronunciation level.

## 1. Introduction

In recent years, one of the hottest study topics in the domains of computer and education has been computer-assisted instruction systems. It has begun to gradually replace teachers to correct, particularly in large-scale language exams, which has become a significant change in the educational field. This type of technology is known as a computer-assisted language learning system [1]. Stress in English is a very essential prosody trait that affects rhythm and intonation, as well as having crucial grammatical and semantic implications. Mastering the stress rhythm correctly plays a crucial role in spoken English expression for language learners [2]. The oral English test now relies primarily on manual marking, which means that scoring is heavily impacted by the raters' psychological and physiological states, and objectivity and fairness of the scoring results cannot be guaranteed [3]. In the sphere of education, English is a critical topic. It will be extremely beneficial to boost students' enthusiasm in studying English, correct students' English pronunciation, evaluate students' academic achievement, and direct students' learning if artificial intelligence is combined with college English skill training [4]. The speech evaluation system is evolving in tandem with the advancement of voice recognition technology. It mostly uses a computer to assess people's pronunciation [5]. For example, the spoken Mandarin test system currently in use can not only score accurately, but it can also considerably enhance efficiency and save personnel [6]. Computer-assisted language learning has progressively begun to suit the diverse needs of language learners, thanks to the rapid development of speech recognition technology and other multimedia technologies. As a result, many researchers have been interested in a pronunciation evaluation system based on speech recognition technology.

Economic globalization has resulted in more regular exchanges between countries around the world today. The fact that English is an Esperanto has also sparked a lot of interest in learning English. People from non-native nations are increasingly keen to enhance their English abilities; in addition to understanding the necessary vocabulary and grammar, improving oral expression ability is also critical. As a result, in non-native countries, the methods of English education and learning have become a hot topic of discussion and research [7]. Oral English training is a crucial component of English instruction. It is difficult for teachers to provide one-on-one oral English instruction due to the large number of students in traditional classroom teaching. After class and examination, oral test questions have become an important link for students to assess their spoken English proficiency [8]. Fortunately, advances in voice processing technologies have made learning English easier and faster. In the realm of voice signal processing, the use of speech technology in language acquisition has become a major trend [9]. It is necessary to develop a system that can assist teachers in intelligently correcting subjective questions in language teaching in order to save time for teachers, save human and material resources for schools, and complete the correction requirements more accurately in order to save time for teachers, save human and material resources for schools, and complete the correction requirements more accurately [10]. Oral Q&A questions can be scored in two ways: from the perspective of pronunciation, and from the perspective of text. Acoustic variables like as pronunciation, frequency, and rhythm are typically used in pure speech scoring [11].

There are two factors to evaluating the level of spoken English in the CALL system based on speech technology. The core method entails identifying phoneme and word faults in learners' spoken language and providing appropriate feedback and solutions. Prosody judgment in spoken language is another factor to consider. For English learners, the latter is more challenging than the former [12]. It is now possible to obtain multidimensional automatic scoring for the open-ended oral language inquiry investigated in this research [13] because to advancements in speech recognition and artificial intelligence technology. This work will combine these mature new technologies with conventional automatic scoring-related technologies, with the goal of using text data after voice recognition as the research object and employing the multifeature fusion method to create a multifeature intelligent correction model. Make intelligent corrections from a variety of perspectives. Artificial intelligence can be used to grade oral expression questions, which appear to have no particular criteria and rely on teachers' subjective judgments. This is important for college English skill training guidance and correction [14]. The examinee's voice is used to do text recognition, with similarity and syntactic features taken from the text and phonetic features extracted from the speech. The automatic correction technology for closed-type oral exam questions cannot be directly transferred to open-type oral test questions due to the distinct principles of correction technology [15]. Because the traditional closed-type question type relies on known text to score speech, while open-type questions have nonunique responses, new text-independent technology is needed to overcome the challenge of open-type oral exam scoring [16]. Speech recognition technology has provided a solution for automatic scoring of large-scale Chinese-English oral translations. The automatic scoring approach not only ensures the correctness of Chinese-English oral translation scoring results, but it also considerably improves scoring efficiency and saves a lot of human, material, and financial resources.

## 2. Related Work

Document [17] examines the automatic correction of spoken language using an automatic composition correction system, and claims that it is possible to correct spoken language using speech recognition technology and a composition correction system. However, the spoken language correction approach suggested in this study has limits, and its scoring dimension is limited, therefore it does not allow for the evaluation of the pronunciation itself. Literature [18] pointed out that in the research of early stress recognition, the recognition system usually extracts some traditional phonetic features, such as sound length, energy, fundamental frequency, etc., and establishes a phonetic model by training linear discriminant function to recognize stressed syllables. Literature [19] puts forward that the functions of intelligent tutor system include building learning environment, communicating with students' emotions, and learning with students. Although the design of intelligent tutor system is called “intelligent” tutor system for evaluating students, it is not smart enough, and it still manages student information and arranges courses according to established rules, which canno't really replace tutors, and has certain limitations. Literature [20] lists the challenges faced by the modeling of simulated human scoring from the process and result levels, and points out that it is impossible to model the evaluation process comprehensively in the field of speech features and speech recognition at present. Literature [21] puts forward that under the dual influence of the abnormal needs of oral English learning and the development requirements of human computer interaction, the pronunciation evaluation system based on language lab recognition technology came into being. It fully integrates the two technologies of CALL and speech recognition to become a more intelligent English learning system, providing learners with opportunities for human computer interaction, and giving a lot of information feedback, completely changing the traditional English learning mode from “teacher teaching” to “student self-learning.”

Literature [22] proposes that in the network environment, the automatic marking system plays a great role in the role orientation of teachers and students, because for non-English majors, their initiative in learning English is not high, and English learning is also very difficult for them. Literature [23] uses deep neural network to improve the robustness of the algorithm and retain the high discrimination of the algorithm, and obtains good experimental results. Although this algorithm is applied to reading questions, we can see that the application prospect of deep learning in oral automatic correction is very good. The combination of deep learning and automatic correction is an important direction for the development of automatic correction system in the future. Through the research on the application of automatic speech recognition system to non-native speech training, literature [24] pointed out that if appropriate methods are used and false pronunciation detection is added, the evaluation system can provide the same evaluation results as human experts. In terms of pronunciation quality evaluation, many studies at home and abroad have proposed various scoring algorithms, and the scoring performance produced by these algorithms has gradually approached the expert scoring level. However, in the research and implementation of pronunciation error detection and feedback information, many systems simply compare the phoneme recognition results with the phoneme correlation results of standard speech. Document [11] the automatic correction system is actually an upgrade of the intelligent tutor system. The intelligent tutor system guides students according to certain rules, and the automatic correction system also scores students' compositions according to the preset scoring rules. Automatic marking system is actually a combination of traditional intelligent tutor system and modern new technology.

## 3. Methodology

### 3.1. Multifeature English Spoken Scoring Algorithm

**Figure 1:**
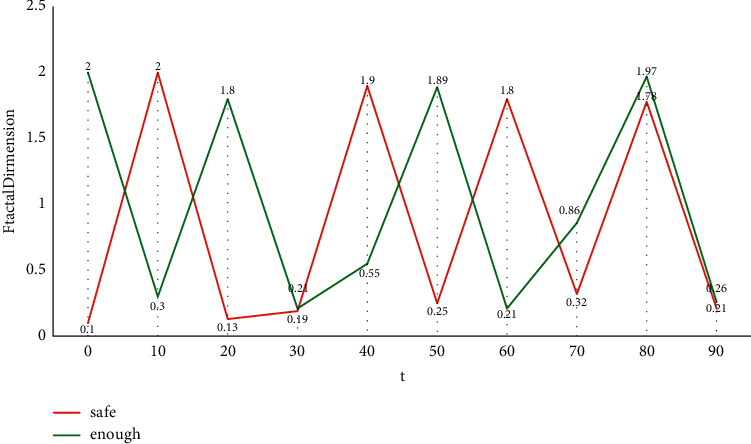
Fractal dimension characteristics of stressed syllables obtained by polymorphic coverage method.

As one of the specific applications of computer-assisted language learning, the overall performance of spoken English scoring system largely depends on speech recognition technology. Multifeature fusion evaluation algorithm includes the evaluation algorithm of a single feature and the algorithm of fusing the evaluation results of multiple features. Because of the instability of the existing automatic speech recognition system, we can evaluate spoken English from this aspect. Multifeature comprehensive evaluation method should have at least the following characteristics: (1) scalability, that is, which features are selected and how many features are selected will not have great influence on the system, and it is easy to add or reduce features. (2) It has nothing to do with the evaluation algorithm of a single feature, that is, changing the evaluation algorithm of a single feature has no effect on the system when the evaluation result form of a single feature does not change. Speech signal analysis is the premise and foundation of speech signal processing. Only by analyzing the parameters that can represent the essential characteristics of speech signals can we use these parameters to process speech communication, speech synthesis, and speech recognition efficiently. And the quality of speech signal processing directly affects the effect of speech recognition. In the early speech recognition system, the isolated word recognition algorithm is the foundation, and its basic principle is to use template matching method for recognition. The fractal dimension characteristics of stressed syllables obtained by the polymorphic method are shown in Figure 1.

**Figure 2:**
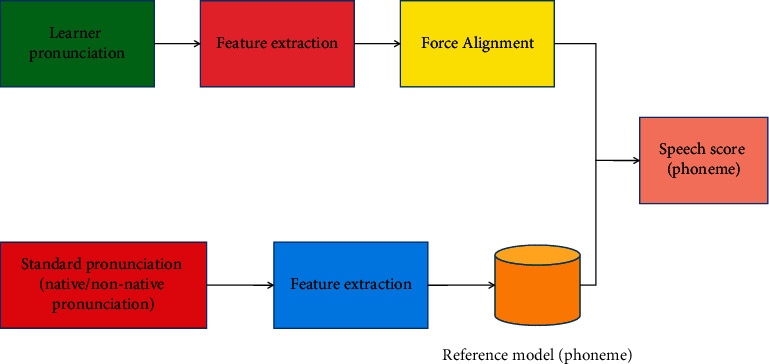
Scoring process based on HMM.

The following aspects are included in feature extraction: (1) energy features. The stationary process processing method and theory are incorporated into the short-term processing of speech signals, considerably simplifying speech signal analysis. (2) Characteristics of duration. The length of each syllable of a multisyllable English word is extracted and compared. Accents are defined as longer syllables. (3) The fundamental features of frequency. Fundamental tone detection is an important technology in speech processing that is frequently utilized in speech recognition, and fundamental frequency is one of the most important prosody parameters in the speech signal. (4) Prediction in linear form. Under the linear prediction technique, the LPC coefficient is a short-term measure of the speech signal. It is the foundation of speech processing and has been used in voice recognition, synthesis, coding, and other applications. The HMM-based scoring method requires pretraining a large number of acoustic models or reference models as a scoring standard, as well as combining the recognition and scoring mechanisms to determine the difference in phoneme pronunciation between the speech to be tested and the standard model and assigning a score. The scoring process based on HMM is shown in Figure 2.

In this method, the number of features can be increased or reduced as long as the weight a I is changed, which meets the first requirement of multifeature comprehensive evaluation method; changing the evaluation result form of a feature, as long as the corresponding quantitative method is changed, does not affect the whole algorithm, so it meets the second requirement of multifeature comprehensive evaluation method. In conclusion, this algorithm has strong scalability and independent of the evaluation model of single feature, and is suitable for comprehensive evaluation of spoken English. The similarity Manhattan distance formula is as follows:

(1) $$\begin{array}{c} D\left(A,B\right)=\sum_{k=1}^{n}\left|a_{k}-b_{k}\right|. \end{array}$$

The formula for calculating the Dice coefficient is

(2) $$\begin{array}{c} D\left(A,B\right)=\frac{2\cdot comm\left(A,B\right)}{leng\left(A\right)+leng\left(B\right)}. \end{array}$$

Conditional probability mean of individual phonemes:

(3) $$\begin{array}{c} P=\frac{1}{n}\sum_{m=1}^{n}P\left(a_{m}|b_{m}\right). \end{array}$$

The loss function is of the form

(4) $$\begin{array}{c} L\left(g_{b},{\hat{g}}_{b}\right)={\left(g_{b}^{\gamma }-{\hat{g}}_{b}^{\gamma }\right)}^{2}. \end{array}$$

The multiscale shape distribution area is obtained as

(5) $$\begin{array}{c} AB^{\left(\varepsilon \right)}=\text{area}\left(F⊕\varepsilon B\right). \end{array}$$

Linear rectification function of the formula is

(6) $$\begin{array}{c} f\left(x\right)=\max\left(0,x\right). \end{array}$$

The log a posteriori probability scoring method based on HMM can not only better reflect the similarity between learners' pronunciation and standard pronunciation, but also reflect learners' pronunciation characteristics from pronunciation units such as phonemes and syllables, and has high stability. Therefore, this scoring method is widely used in relevant recognition systems or learning systems.

### 3.2. Design of Multifeature Intelligent Scoring Model

**Figure 3:**
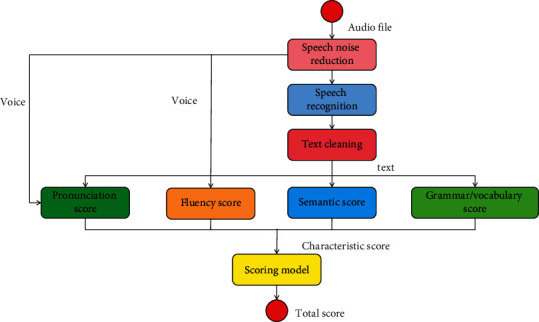
Multifeature intelligent scoring model.

The whole scoring system includes three parts: speech recognition, feature extraction, and linear regression. The similarity features and syntactic features in feature extraction are extracted from the text after speech recognition, and the similarity features need to be compared with the reference answers. Automatic scoring of oral translation is mainly from two perspectives. One is to directly process the speech of oral translation and score oral translation directly; another point of view is to first convert the speech of oral translation into text, and indirectly score oral translation through text processing. The design purpose of the model does not focus on how to effectively recognize learners' speech, but how to improve the accuracy of system evaluation, reduce the misjudgment rate of pronunciation error detection, and whether the scoring accuracy of the system is improved when the average pronunciation scoring level is adopted under the same acoustic model. In the multifeature fusion evaluation system, the system only needs to recognize the input once, then evaluate the recognition results of multiple features respectively, and use the evaluation results of multiple features to give the final evaluation results. The multifeature intelligent scoring model is shown in Figure 3.

**Figure 4:**
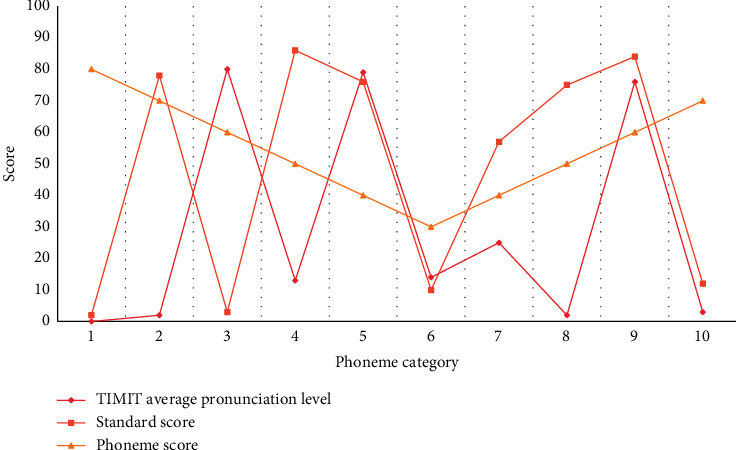
Comparison between score results and average pronunciation level.

Multifeature fusion evaluation system includes four parts: speech recognition, grammar expansion, single feature evaluation, and result processing. In order to make our feature extraction work smoothly and improve the accuracy, we also designed three data processing modules in the scoring model: (1) speech noise reduction module, (2) speech recognition module, and (3) text cleaning module. The training data is input into the neural network, and then the neural network is used for continuous training to train an intelligent scoring model. Then the test data is input into the intelligent scoring model to obtain the preliminary results of intelligent scoring, and the results are compared with the teacher's scores. Then, the model is feedback optimized, the model is improved, and finally the intelligent scoring model is determined. According to the statistical threshold of the corresponding phoneme, it is determined whether the learner's pronunciation is wrong, and the comparison between the score result and the average pronunciation level is shown in Figure 4.

**Figure 5:**
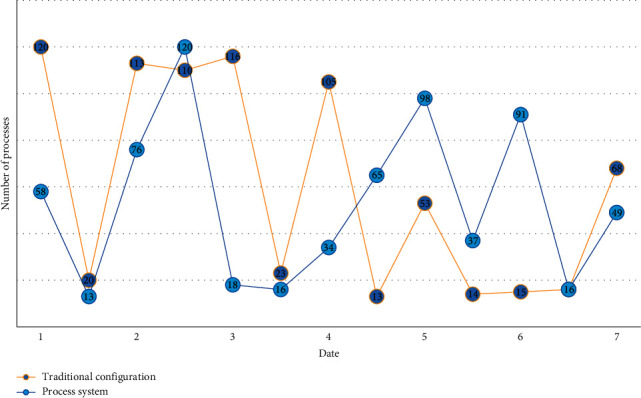
Comparison of the number of process runs.

Each extended new word's phonetic symbols are paired with the acoustic model and added to the system's lexicon. In this approach, the system can determine whether the oral practitioner pronounces the erroneous sound if the recognition result of the input speech is the expanded new word. When creating the system, the major design objectives should always be the system score, error prompt, and correction feedback. Simultaneously, we should consider the friendliness of the interactive interface between the system and users, as well as learning mode innovation, as additional objectives, in order to avoid reducing learning interest or efficiency due to boring learning forms or content, and to improve the system's operability. The statistical data of the operation process of the traditional configuration method and process system within one week are randomly intercepted, as shown in Figure 5.

First, feature extraction is performed on the dataset, and then these feature data together with the data labels are input into the neural network for supervised learning. After a period of learning and training, a classification model is finally generated, and we can use this model to extract features from the new spoken audio, and then input the features for scoring. The whole scoring process of the system is carried out from two aspects. On the one hand, the system itself needs to extract features and creates models for the selected standard corpus. At the same time, in order to eliminate the wrong scoring caused by the differences between standard pronunciation speakers, it also processes the standard pronunciation correspondingly, and selects the average of all phonemes as another reference factor to judge whether learners pronounce incorrectly or not. On the other hand, when learners input speech, the system will extract the features of their pronunciation through the speech recognizer, divide the feature values into phoneme-level scoring units, remove noises and noises, compare them with the standard reference model after forced alignment, and use the scoring algorithm in Chapter 3 to get the corresponding phoneme scores, thus completing the preliminary scoring of the system.

## 4. Result Analysis and Discussion

### 4.1. System Implementation

**Figure 6:**
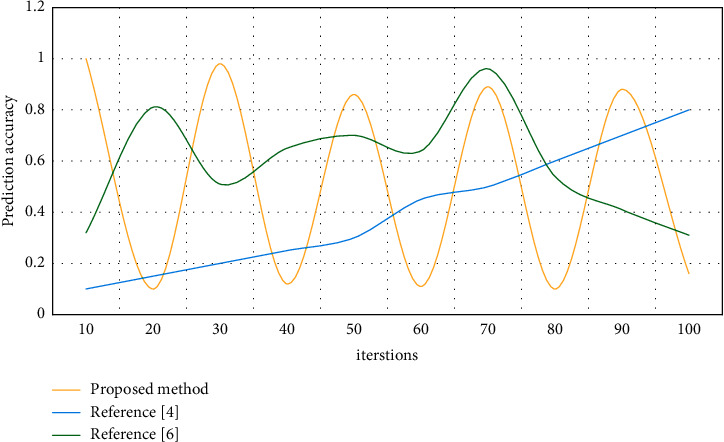
Comparison of detection performance.

At first, the system will record the voice input by the user. After the user input is completed, the user input will be recognized by voice, and the result of the recognition will be expanded by grammar. Then, the linking and confusing sounds will be evaluated separately. Finally, the evaluation results of the two will be synthesized and the comprehensive evaluation results will be given. Automatic scoring performance is measured by correlation coefficient. The larger the correlation coefficient, the more consistent the scoring trend of experts on samples. The interface layout of English automatic scoring system is mainly divided into two parts. One is the selection and input of learners' pronunciation content, and the other is the feedback display of the system to users' pronunciation. Therefore, the realization of other main functions of the system, including speech recognition, is also realized in two parts. A linear criterion is used to distinguish the two categories optimally. The optimization criterion always increases the distance between classes and reduces the distance between classes. Most of the discriminant information can be obtained from the transferred feature space. The result processing part first quantifies the evaluation results of a single feature, and then synthesizes the quantized results to obtain the evaluation results of multifeature fusion. Finally, this chapter gives the realization method of the system. Users can choose the courses and exercises they want to learn. The system gives a comprehensive evaluation of the user's pronunciation, and marks the specific situation of the user's pronunciation and the areas that need improvement with special colors. Realize the pronunciation error detection of spoken English, test the detection performance, and get the results as shown in Figure 6.

Before comparing the scores, we must first segment the candidates' voice. Because the examinee has frustrations, repetitions, and other phenomena in oral expression, we are unable to 100% correspond the text and audio after speech recognition in the time dimension. Therefore, we need to segment the audio and try to align the pronunciation audio of standard words with the examinee's voice in the time series. If the user's pronunciation is not accurate enough, the system will refuse to evaluate and ask the user to learn again. After recording, learners can input their own pronunciation into the system, play back their previous pronunciation by using the user play function key, and get the score of previous pronunciation and corresponding correction opinions by using the scoring function key. Of course, if you cannot understand the problems of your pronunciation through scores and correction opinions, you can also compare the differences between your pronunciation and standard pronunciation to improve your pronunciation. You can only use standard playback. The whole process of system scoring mainly includes feature extraction, forced alignment, HMM-based posterior probability score calculation, mispronunciation judgment, correction feedback, and other processes. According to the needs of the system, the corresponding technical links are modified to realize the final scoring process. Learners can choose to play the standard pronunciation, and at the same time hear the pronunciation of the standard pronunciation, they can also see the score of the standard pronunciation in the right area of the scoring box, so that they can more intuitively see the differences between their own pronunciation and the standard pronunciation. At the same time, the expert opinion database will determine the final correction opinions according to learners' scores, standard pronunciation scores, and average pronunciation level of standard pronunciation.

### 4.2. Result Analysis

**Figure 7:**
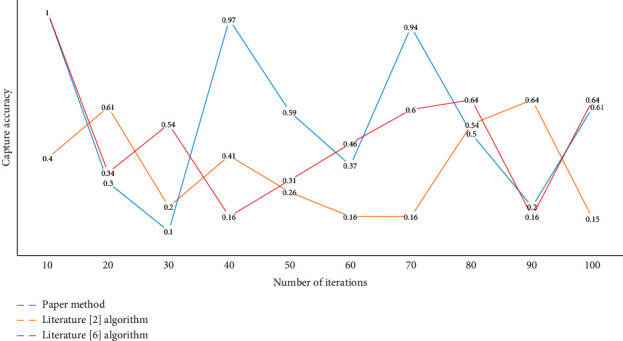
Comparison of capture accuracy.

After using the average level as a reference, the judgment threshold has changed correspondingly, and the accuracy rate of wrong judgment of the system has been improved, which leads to the improvement of the accuracy rate and the reliability of feedback information.  Figure 7 shows the results of comparing the accuracy of error capture of several approaches of spoken English pronunciation.

Because the learner's voice is first compared with the standard voice, the average level will be used for comparison when there is a large gap, so as to ensure that the learner's voice is infinitely close to the standard voice in terms of pronunciation level without pronunciation error and misjudgment, which also meets the design requirements of similar systems. The free English composition automatic correction online service based on corpus and cloud computing can instantly generate the scores, comments, and content analysis results of students' compositions by calculating the distance between students' compositions and standard corpora, which can provide multidimensional scoring results. Artificial intelligence can play the teaching role of constructivism, because artificial intelligence can analyze data in a short time, so it can find teaching resources that are conducive to learners' characteristics and in line with learners' cognitive style. Artificial intelligence can store and analyze a large amount of learner characteristic data. Through the analysis of learners' learning style and cognitive level, teachers can understand learners' advantages and disadvantages, make teachers timely and accurately adjust teaching strategies and teaching methods, and make learners more acceptable. Therefore, artificial intelligence can better guide teaching practice.

After the system adopts an average pronunciation level based on standard speech as the error judgment standard, the accuracy of error detection has been improved to a certain extent. It is worth noting that since the system adopts the corpus of foreign pronunciation, it is possible to deviate from the normal value only from the score given to learners by the system, but when the same acoustic model is used as the system score. The verification of error detection accuracy is not affected, because the average pronunciation level used here is based on standard pronunciation as the source data. It is assumed that when the Chinese pronunciation corpus is used as the standard pronunciation for scoring and error judgment, the score is closer to the learners' real level, but the error and misjudgment caused by individual differences in standard pronunciation is still inevitable. Based on the multifeature fusion evaluation algorithm, the training and testing results show that the performance of the algorithm is good and the algorithm shows good stability under different weights. The automatic scoring system of spoken English is realized, with emphasis on the recording function, scoring process, and the realization process of expert correction feedback. The experimental data are analyzed by two error judgment methods: standard pronunciation score and average pronunciation level, and adding average pronunciation level to judge. The experiment shows that the method proposed in this study can effectively reduce the misjudgment rate, and the running performance of the system itself is stable, which basically meets the practical application standards of this kind of system.

## 5. Conclusion

The salient points of this study are: first, try to take speech recognition text as the research object to score the oral test; secondly, the similarity feature is improved, and a keyword coverage based on editing distance is proposed, and these two features are better than those obtained by traditional methods; thirdly, multiangle feature extraction and multifeature fusion in linear regression system. Fourth, select features and delete redundant features. Although the accuracy rate of speech recognition has been claimed to be over 95% in official documents, in the actual process, due to the test environment, the influence of students around and the noise nearby, the output result of speech recognition will be affected. The speech features are extracted by direct analysis of speech signals, the similarity features and syntactic features are extracted by analysis of the text after the speech signals are converted, and the automatic scoring system is obtained by using multiple regression analysis model. The corresponding evaluation algorithm is established, and features are added into the multifeature fusion evaluation system. Comprehensive evaluation system of spoken English, which integrates a large number of features, will be the future development direction. At present, the automatic scoring technology of open spoken questions is deeply studied and compared. Through feature fusion, Rank Net neural network maps the complex nonlinear relationship among features, and establishes the recognition model of stressed syllables in words and stressed sentences, which can accurately recognize stressed syllables in words and stressed sentences. This study summarizes the valuable experience of predecessors' work, innovatively applies and improves the key technologies, integrates and improves the latest technical schemes at present, puts forward and realizes a new open oral correction model, innovates the method of voice feature extraction, and improves the dimension of open oral scoring.

## Data Availability

The data used to support the findings of this study are available from the corresponding author upon request.
